# Discussing the role of circular RNA in the pathogenesis of non-alcoholic fatty liver disease and its complications

**DOI:** 10.3389/fendo.2022.1035159

**Published:** 2022-11-02

**Authors:** Melissa Yepmo, Jean-Baptiste Potier, Michel Pinget, Anastasia Grabarz, Karim Bouzakri, Aurore Dumond Bourie

**Affiliations:** ^1^ Centre européen d’étude du Diabète, Unité Mixte de Recherche de l’Université de Strasbourg « Diabète et Thérapeutique », Strasbourg, France; ^2^ ILONOV, Strasbourg, France

**Keywords:** NASH, NAFLD, circRNA, liver, crosstalk, hepatocellular carcinoma, insulin resistance

## Abstract

Circular RNAs (circRNAs) are class of non-coding RNA, which are characterized by a covalently closed loop structure. Functionally they can act on cellular physiology, notably by sponging microRNAs (miR), regulating gene expression or interacting with binding protein. To date, circRNAs might represent an interesting, underexploited avenue for new target discovery for therapeutic applications, especially in the liver. The first characteristic of non-alcoholic fatty liver disease (NAFLD) is hepatic cholesterol accumulation, followed by its advanced form of the affection, nonalcoholic steatohepatitis (NASH), due to the occurrence of lobular inflammation, irreversible fibrosis, and in some cases hepatocellular carcinoma (HCC). Therefore, studies have investigated the importance of the dysregulation of circRNAs in the onset of metabolic disorders. In this review, we summarize the potential role of circRNAs in the development of metabolic diseases associated with the liver such as NAFLD or NASH, and their potential to become therapeutic strategies for these pathologies.

## Introduction

Metabolic associated liver diseases, such as non-alcoholic fatty liver disease (NAFLD) have been emerging these last decades all around the world due to the rising of type 2 diabetes, western diets and sedentary lifestyle (1). NAFLD is a chronic liver disease that affects 25% of adults worldwide and is associated with insulin resistance, *de novo* lipogenesis, dyslipidemia, obesity and a higher cardiovascular risk. The pathophysiology of NAFLD is characterized by the presence of steatosis, defined by the accumulation of lipid vacuoles in more than 5% of all hepatocytes (2). In addition to metabolic syndrome, type 2 diabetes and obesity, numerous NAFLD risk factors exist such as gender, ethnicity and genetic predispositions (3). Among these predispositions, genetic polymorphism such as those related to variants of the genes PNPLA3 and TM6SF2 have become widely studied and have proved their crucial association with the onset of NAFLD during the last few years (4).

In itself, NAFLD can remain clinically silent and undetected for most patients (5). However, in 25% of the cases, NAFLD can progress towards a more severe affection: non-alcoholic steatohepatitis (NASH) (6). NASH is a chronic liver disease whose pathophysiology includes the presence of lobular inflammation, hepatocyte ballooning, oxidative stress, and fibrosis (7) and can be related to more severe outcomes, such as cirrhosis, acute liver failure (8), liver transplantation (8) or hepatocellular carcinoma (HCC) (9, 10). In this context, the homeostasis of the hepatic microenvironment is crucial in the progression of NAFLD to NASH. Three main cell type are implied in this process: hepatocytes, hepatic stellate cells (HSC) and liver macrophages. Hepatocytes are the hub of liver metabolic functions (11) and the first liver injured cell type in NAFLD, through steatosis (12). Resident and circulatory liver macrophages are widely responsible for the onset and progression of liver inflammation during NASH (13), even if their decisive role beyond inflammation has been emerging lately (14). Finally, hepatic stellate cells are a quiescent cell type that mainly store vitamin A in healthy individuals (15). However, in the context of liver injury and NASH, they can become activated, transdifferentiate into myofibroblasts and acquire a pro-fibrotic and pro-inflammatory phenotype, leading to the development of liver fibrosis (16).

Despite the multiplication of potential new drug candidates and clinical trials, there is still no approved treatment for NASH, justifying the urge of a finding (17). The last few years have been critical for the elucidation of new pathways involved in liver diseases pathophysiology, thanks, notably, to the democratization of new breakthrough methods in the field of metabolomics, proteomics and transcriptomics, opening the way to new therapeutic hopes (18–20). Among these new emerging subjects, non-coding RNAs and their pleiotropic roles in chronic liver diseases has been widely studied (21, 22). Many types of non-coding RNAs exist including long non-coding RNAs (lncRNAs), transfer RNAs (tRNAs), micro RNAs (miR), ribosomal RNAs (rRNAs) and circular RNAs (circRNAs) and can represent promising therapeutic agents (23).

Circular RNAs were first discovered in 1976 in plant viroids, observed by electron microscopy (24). They were then identified in eukaryotes in 1979 in the cytoplasmic fraction of eukaryotic cell lines (HeLa cells) (25). Their structure is characterized by a covalently closed loop and lacks polyadenylation at the 3’ and 5’ end, protecting them from exonucleases and strengthening their stability (26). Recent studies have shown that they are generally generated by reverse splicing or exon skipping of pre-messenger RNAs (mRNA) (27). For a long time, due to technical limitations in research, circRNAs were considered as abnormal splicing products from pre-mRNA and their functions were not yet fully understood (28). The last 10 years resulted in major discoveries of the functional role of circRNAs. With the use of high-throughput sequencing and bioinformatics, increased numbers of circRNAs have been identified in eukaryotes, whether in humans (29), animals or plants (30) and the number of published studies on circRNAs has increased exponentially. Their main role as microRNA (miR) sponges has been highlighted (31), fine tuning the transcriptome’s activity. MicroRNAs (miR) are small non-coding RNAs that fine-tune gene expression at the post-transcriptional level, by binding to the 3′ untranslated regions of target mRNAs and inhibiting their expression. Cytoplasmic circRNAs can contain miR binding sites in their sequences and therefore sequester these small RNAs, preventing the interaction with specific mRNA targets (32, 33). As a result, circRNAs represent one of the most remarkable molecules in RNA biology (34), constituting an important part of the cellular transcriptome (31, 32). To date, there are three main classes of circRNAs located in different cellular compartments. Most of them have a cytoplasmic localization (29), in particular with the Exonic circRNAs (EcircRNAs), which have a sponge role of inhibiting the action of target microRNAs (31). There are also the Exon-intronic circRNAs (EIcircRNAs) and Intronic circRNAs (IcircRNAs), which are found in the cell nuclei and have a role in the regulation of gene expression (33).

Importantly, these circRNAs are related to proliferation, invasion, migration, angiogenesis, apoptosis, and metastasis of cells in liver diseases and act as oncogenic agents or suppressors and are linked to clinical manifestations. In this context, circRNAs gained importance in the discovery of new potential therapeutic targets, particularly in liver-associated metabolic diseases. Thus, we will review their role in the etiology of NAFLD and NASH.

As of today, most of the studies about circRNAs and the liver have been focusing on hepatocellular carcinoma (35) and few studies concern their contribution to NASH development, in this review, we will discuss the role of different circRNAs and their implication in NASH, as well as their potential connection with biological mechanisms linked to the onset of NASH.

## CircRNAs’ involvement in NAFLD pathology

Non-alcoholic fatty liver disease (NAFLD) is one of the most common liver diseases in developed countries, with the fastest growing in the USA, France and the UK. A strong increase is also observed in developing countries and worldwide in general (36, 37). In 20–30% of cases, NAFLD slowly progresses to non-alcoholic steatohepatitis (NASH), fibrosis, cirrhosis and hepatocellular carcinoma (HCC). NAFLD’s main hallmark is the accumulation of triglycerides in the liver (38).

### CircRNAs in lipid metabolism

Recent studies have reported that circRNAs are important regulators of hepatic steatosis, the main characteristic of NAFLD (39). However, the roles and mechanisms of circRNAs in NAFLD are still poorly understood (40). In different studies, RNAseq techniques as well as bioinformatics analysis has allowed to map for the first time 93 dysregulated circRNAs in NAFLD mice. As a result, 57 overexpressed and 36 underexpressed circRNAs were identified as potential cellular biomarkers in the pathogenesis of NAFLD (37). Furthermore, abnormal lipid metabolism in the liver is often accompanied by disordered expression of circRNAs (41). For example, JAK2/STAT5 pathway plays an important role in NAFLD by regulating the growth hormone pathway and their aberrant expression can lead to abnormal lipid metabolism (42). It has been identified that circSCD1 stimulates protein expression of JAK2 and STAT5 and ultimately affects the pathogenesis of NAFLD by promoting hepatic steatosis *via* the JAK2/STAT5 pathway (43).

Some studies also highlighted the effects of circRNA_0000660 on cellular lipid accumulation and its target gene in AML-12 hepatocytes. CircRNA_0000660 is a specific antagonist of miR_693 that directly targets Igfbp1 (44). Indeed, knockdown of circRNA_0000660 has been shown to increase lipid accumulation through the downregulation of Igfbp1 level (45).

Recent studies highlighted the crosstalk between circRNA hsa_circ_000313 with miR-6512-3p-PEG10 by identifying its role in the physiopathogenesis of NAFLD, but further studies are needed to deepen its beneficial role and to determine if this circRNA could become a potential biomarker for the disease (46).

All these results have shown that circRNAs have a huge involvement in the development of lipid disorder through different signaling pathway and can be potential blood biomarkers to detect an early stage of NAFLD.

### CircRNAs in autophagy

Autophagy, also referred to as “self-eating”, is a process found across many species in the eukaryotes. It involves the delivery and degradation of cytoplasmic materials by lysosomes and plays a prominent role in cell survival, differentiation, development, and homeostasis by controlling cytoplasmic physiology through maintaining energy balance and the removal of misfolded proteins, damaged organelles, and lipid droplets (47, 48).

Alterations in the cell autophagic machinery have been implicated in several disease conditions, including non-alcoholic fatty liver disease and non-alcoholic steatohepatitis (49). Accumulating evidence suggest that maintaining intact autophagy pathways may represent a valuable target in NAFLD, because of its anti-steatogenic properties in hepatocytes (lipophagy) and its beneficial effects in inhibiting the progression to NASH through the hepatoprotective effects of mitophagy in hepatocytes and its anti-inflammatory properties in macrophages (50).

For example, circRNA_002581 has been described to play a role in hepatic autophagy by sponging mir-122. Studies already showed the involvement of this mir-RNA, thereby a decrease of mir-122 was observed with an increase of hepatic triglyceride accumulation and a total stop of fat oxidation in NASH patients through the expression of cytoplasmic polyadenylation element-binding protein 1 (CPEB1) (51). As a result, endogen CircRNA_002581 have a negative role by sponging mir-122 and thus leads to an increase of CPEB1. This dysfunction act on the autophagy by a decrease through PTEN/AMPK/mTOR signaling pathway and increase NAFLD progression (52). Thus, knockout of CircRNA_002581 leads to partial autophagy restoration. Finally, these results have shown that the circRNA_002581–miR-122–CPEB1 axis has therapeutic potential in NASH *via* autophagy (52).

**Figure 1:**
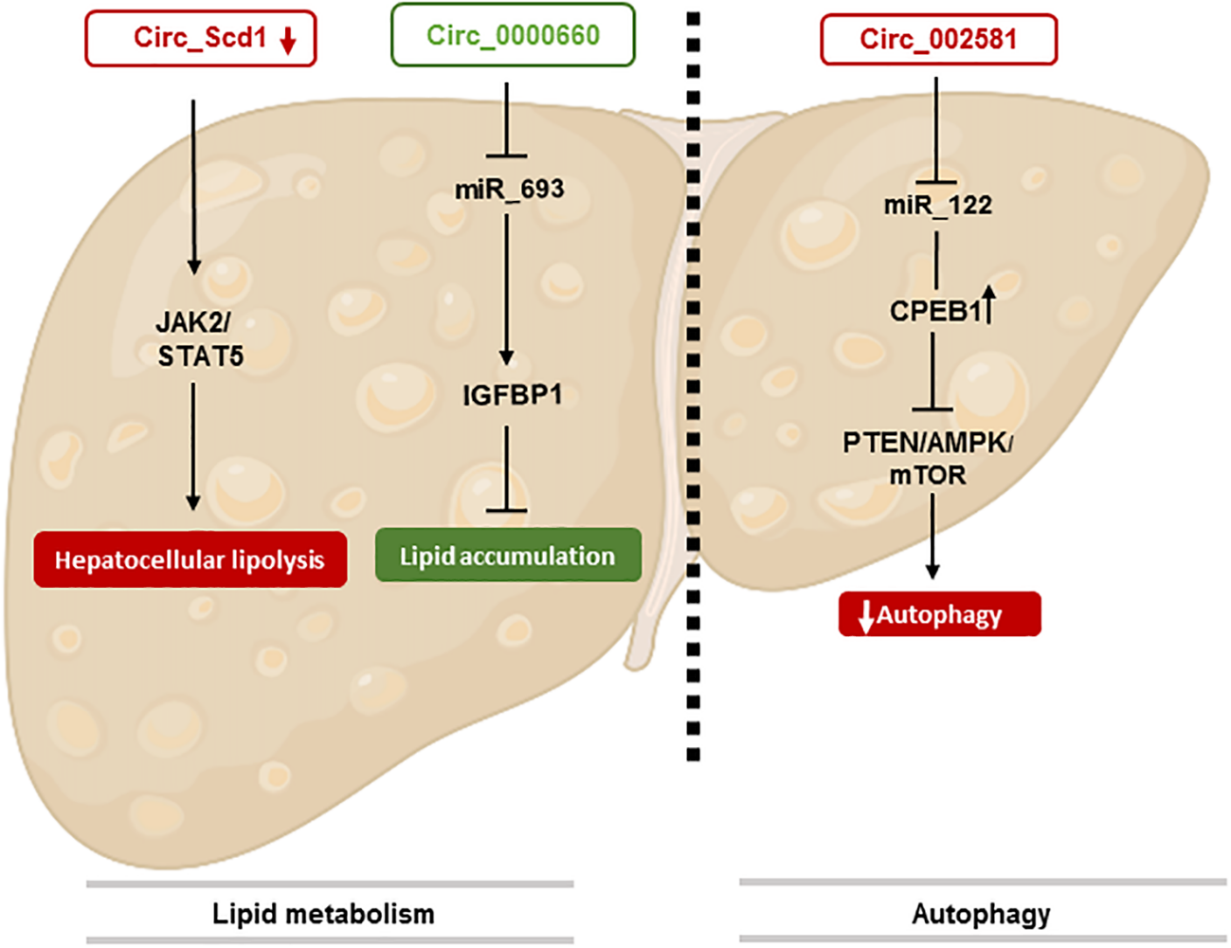
CircRNAs involved in NAFLD stage. This figure represents the effect of circRNAs in lipid metabolism through different pathways. The identification of circRNA involved in autophaghy is also shown.

The role of circRNAs in the regulation of autophagy in NAFLD is not yet clearly described but nevertheless represents an important key regulator and therapeutic target (**Figure 1**).

## CircRNAs and their impact on the pathophysiological features of NASH

**Figure 2:**
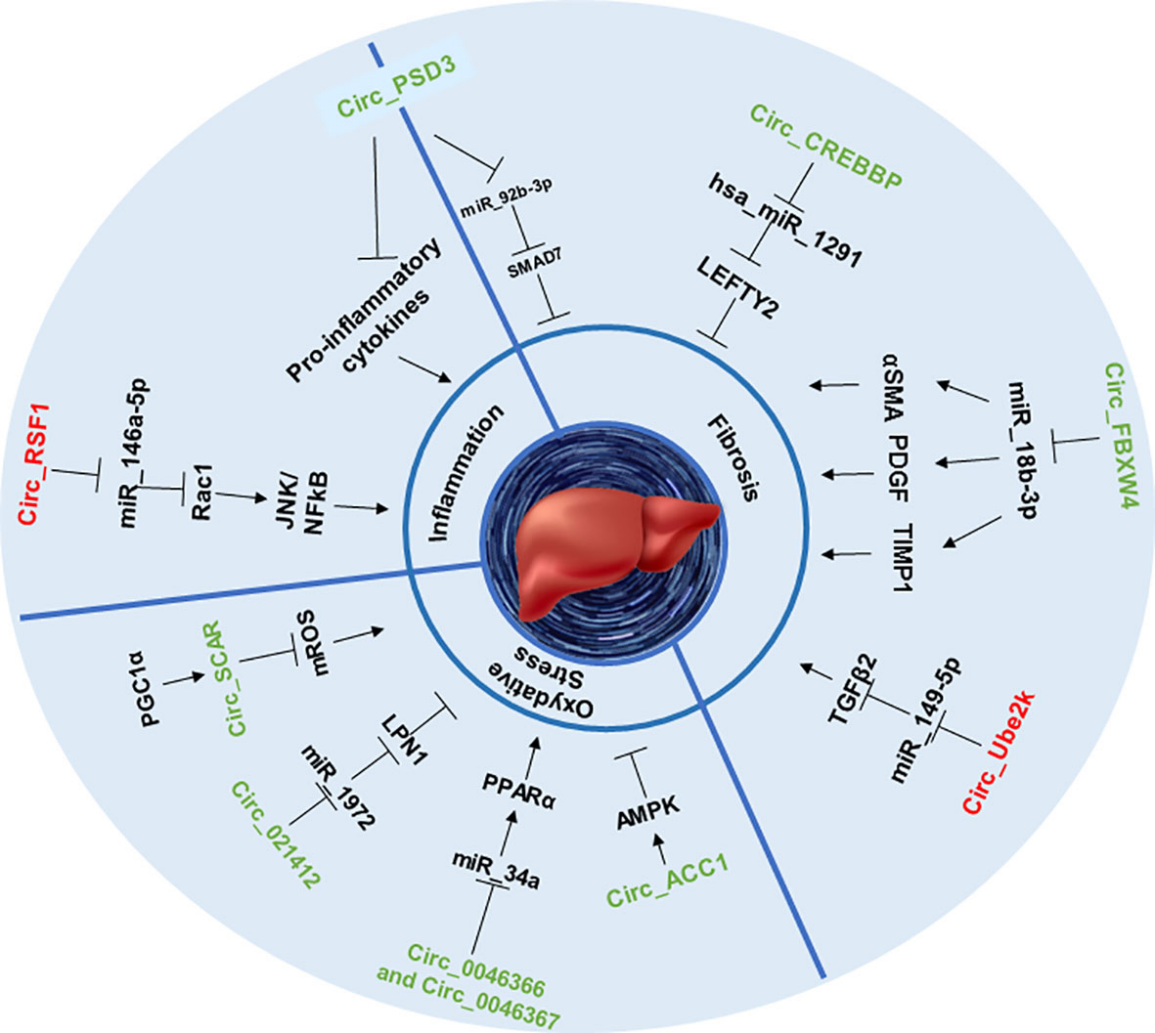
CircRNAs involved in NASH stage. This figure represents the effect of circRNAs in inflammation, fibrosis and oxidative stress through sponging specific miRNAs that can upregulate or downregulate specific proteins and signaling pathways.

Among all patients suffering from NAFLD, 25% to 44% of them will develop the more severe form of this disease: NASH (53). A theory aiming to explain the relationship between NAFLD and NASH has emerged during the last decade: the “two-hit” hypothesis. According to this theory, the “first hit”: NAFLD, as previously described is a hepatic metabolic disorder with increased *de novo* lipogenesis, unpaired β-oxidation and steatosis. Those first injuries will enhance the susceptibility of the liver to progress to the “second hit”: NASH, characterized by lobular fibrosis, inflammation and oxidative stress (54). Thus, in the next part, we will focus on the impact of circRNAs in the onset of the NASH hallmarks (**Figure 2**).

### CircRNAs as key regulators of liver fibrosis

Fibrosis remains the most important parameter defining the severity and outcome of patients with NASH (55), especially regarding the evolution of the disease toward cirrhosis and HCC (10). Liver fibrosis can be measured by staging, from F0, indicating no fibrosis to F4 being the most advanced form of fibrosis, namely cirrhosis (56). In this context, the activated HSC will secrete significant amounts of collagen and extracellular matrix in the hepatic microenvironment, inducing an abnormal level of nonfunctional scar tissue, replacing progressively healthy tissue, leading to cirrhosis and diminished liver function. Among all NASH hallmarks, liver fibrosis is undoubtedly the one for which circRNAs have been the most widely studied. Induction of NASH with repeated doses of carbon tetrachloride (CCl4) in mice is a widely used *in vivo* model of liver fibrosis. Using this approach, several authors highlighted the role of circRNAs in the onset of liver fibrosis.

Some circRNAS, for example, circPSD3 was shown to be downregulated in HSC and in the liver of fibrotic mice. By sponging miR-92b-3p, circPSD3 promoted the expression of Smad7, a protein known as an antagonist of the pro-fibrotic TGF-beta pathway (57, 58). Thus, circPSD3 reduced HSC proliferation and activation and alleviated the accumulation of collagen as well as the expression of pro-fibrotic genes *in vitro* and *in vivo* (59). Using similar methods, another circRNA, circCREBBP, was shown to prevent CCl4-induced fibrosis progression by sponging hsa-miR-1291, thereby activating the expression of LEFTY2, a protein from the TGF-β family with anti-fibrotic properties (60, 61). Again, circFBXW4 showed a similar anti-fibrotic capacity by targeting miR-18b-3p, an inducer of the expression of fibrogenic factors such as α-SMA (62), PDGF (63) and TIMP-1 (64) in HSCs (65).

However, the properties of circRNAs are not always beneficial, as recent studies highlighted their role in the promotion of fibrosis as well. For example, by sponging miR-149-5p, circUbe2k induced the upregulation of the TGF-β2 pathway, resulting in the exacerbation of CCl4-induced liver fibrosis (57, 66).

Taken together, it appears that circRNAs can exert bimodal effects on the onset of liver fibrosis, depending on their sponging capacities and miR targets.

### Implication of circRNAs in the pro-inflammatory process within the liver

Inflammation is one of the key features that characterize the progression of NAFLD to NASH. This process is often coming from change inside the hepatic microenvironment following pro-inflammatory cytokines’ circulation and macrophages’ infiltration, leading to lobular inflammation (67). Lobular inflammation is a common hallmark of NASH caused by an accumulation of immune cells inside the hepatic lobules, due to chronic inflammation, the anatomic units of the liver (68, 69). In this context, inflammatory cytokines can be secreted by various cell types, such as activated hepatic stellate cells (70) or M1 macrophages (71). CircRNAs are showing potential implications in these phenomena.

As discussed previously, circRNAs can impact HSC activation in several ways and could therefore be involved in the onset of inflammation in the context of NASH. For example, HSC activation can be obtained by challenging cells with irradiation, however, further studies are necessary to determine if irradiation challenge is physiologically relevant to mimicking the onset of NASH. In this context, it was shown that irradiation of the hepatic stellate cell line LX2 leads to the upregulation of circRSF1 and its sponging of miR‐146a‐5p. CircRSF1, by inhibiting miR‐146a‐5p-downregulation of Rac1, a protein involves in liver injury (72), increased pro-inflammatory pathways such as JNK (73) or NF-κB (74) and the secretion of pro-inflammatory cytokines such as TNFα, IL-1β or IL-6 in LX2 cell line (75). In another previously cited study (59), the authors found that the expression of circPSD3 was highly downregulated in the liver of mice treated with CCl4 and also within tissues of humans suffering from hepatic fibrosis. Infection of the animals with an adeno-associated vector carrying circPSD3 (AAV8-circPSD3) alleviated the production of pro-inflammatory cytokines by activated HSC and the infiltration of inflammatory cell infiltration (59), suggesting the positive role of this circular RNA in the prevention of liver inflammation.

In another recent study, circRNA_1639 was found to act as a sponger of the miR-122 within the liver and hepatic macrophages, triggering the inflammatory response (76). However, these findings were concerning mice liver in the context of alcoholic liver injury and could therefore be less relevant to NASH injury, thus, further studies are needed to confirm the same role of circRNA_1639 in the specific mechanism of NASH inflammation.

Since few studies have been focusing on this topic, it would be particularly interesting to study the role of circRNAs in the activation or migration of liver macrophages in the context of NASH, independently of HSC activation.

### CircRNAs in oxidative stress

The pathophysiology of steatohepatitis and its progression are also influenced by multiple environmental and genetic factors, in which oxidative stress most likely plays a primary role as a starting point for hepatic and extra-hepatic damage (77). Moreover, numerous studies have indicated that this pathology is characterized by the presence of mitochondrial dysfunction (78). Indeed, circACC1 promotes fatty acid β-oxidation by facilitating the assembly, stability, and activity of the AMP-activated protein kinase (AMPK) holoenzyme in LO2 hepatocytes. Loss of AMPK-binding activity of circACC1 resulted in impaired mitochondrial fatty acid β-oxidation and increased steatosis in LO2 cells (78, 79).

CircRNA_021412 is increasing fatty acid β-oxidation and decreases the synthesis of triglyceride in HepG2 hepatocytes through the miR‐1972/lipin1 (LPIN1) pathway (44). As a result, downregulation of CircRNA_021412 plays an important role in the induction of steatosis-related genes *via* PPAR alpha activation. Indeed, after high fat stimulation there is a decrease in circRNA_021412 inhibition of mir-1972, so a reactivation of mir-1972 leading to the down-regulation of LIPIN 1 level, which will induce the activation of steatosis-related genes (80, 81).

CircRNA_0046366 and circRNA_0046367 promoted fatty acid β-oxidation in HepG2 cells (44, 82). They both prevent the binding of miR-34a to PPARα and alleviate hepatic steatosis by restoring the lipid metabolism pathways and genes (83, 84).

Recently, Zhao et al. have demonstrated that, in healthy conditions, PGC1-α activates the binding of circ_SCAR on ATP5B, which inhibits mPTP’s interaction with CypD in the mitochondria and so the activation of mROS (85). In a patient with NASH, lipid-induced ER stress reduces PGC-1α-mediated circRNA_SCAR expression and thus increases the production of mROS-induced pro-inflammatory activation of liver fibroblasts. Thus, circ_SCAR restoration in NASH may be a potential therapeutic target (85).

## CircRNAs and hepatocellular carcinoma

**Figure 3:**
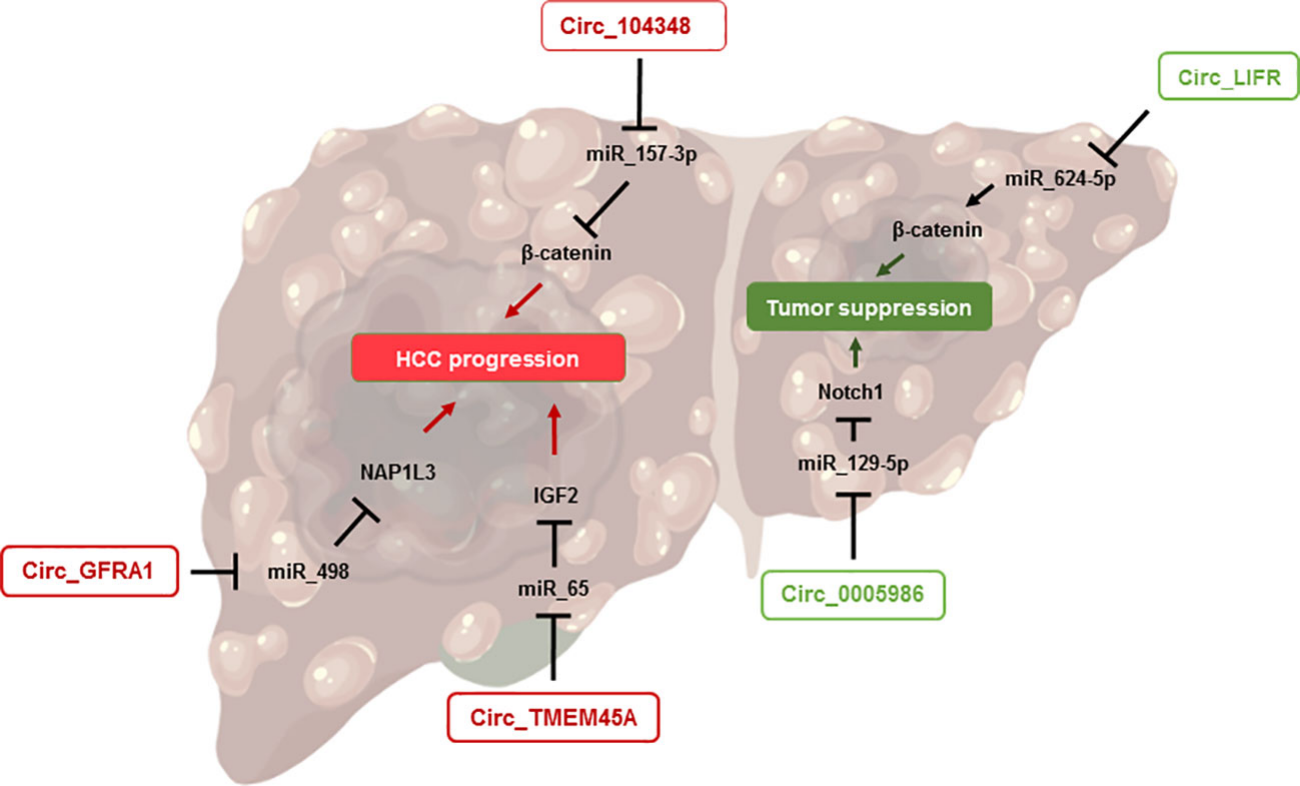
CircRNAs involved in HCC onset. This figure shows the implication of circRNAs in the pathophysiology of HCC. The right side represents those promoting tumor suppression while the left side highlights the circRNAs exacerbating HCC progression.

As previously stated, patients suffering from NASH are at higher risks of developing hepatic complications, such as liver failure, infectious diseases or hepatocellular carcinoma (55). Hepatocellular carcinoma is the most common form of liver cancer, representing up to 90% of hepatic tumors (86). HCC is often described as the final stage of NASH and occurs predominantly among patients suffering from the late stage of fibrosis: cirrhosis (10, 87). The pathophysiology of NASH-related HCC is not yet fully understood, but several approaches do exist. It is widely accepted that the perturbation of the hepatic microenvironment occurring in patients with severe NASH largely contributes to DNA mutations and abnormal metabolic homeostasis, as well as uncontrolled liver regeneration and scarring, and thus to the onset of HCC (10) (**Figure 3**).

During the last decade, various biological functions of circRNAs in HCC have been studied. For example, circLIFR has been highlighted as a tumor suppressor in HCC, by sponging miR-624-5p and downregulating the β-catenin signaling pathway (88), which acts as an oncogenic signal through its ability to induce the transcription of several genes related to proliferation in hepatocytes (89). Another circRNA, circ_0005986, has been suggested as a promising candidate in the study of HCC, both as an inhibitor of carcinogenesis and as a prognostic biomarker. For the first aspect, circ_0005986 acts by inhibiting liver cell proliferation through the sponging of miR-129-5p and the regulation of Notch1, whose signaling regulates growth, apoptosis, and cell differentiation (90). Furthermore, low levels of circ_0005986 in HCC tumors were correlated with larger tumor size, while higher levels were associated with improved survival (90, 91), suggesting a promising role of this circRNA as an HCC prognostic biomarker.

Some circRNAs have been identified lately as promoters of hepatocellular carcinoma as well. For instance, circTMEM45A was upregulated in patients with HCC, and its expression was correlated with the severity of the disease. By using *in vitro* and *in vivo* approaches, the authors showed that circTMEM45A could promote the progression of HCC by sponging miR-65, resulting in the activation of the IGF2 pathway and ultimately to enhanced cell proliferation and cancer development (92). Similar results were obtained for CircRNA circ_GFRA1, which modulates the miR-498/NAP1L3 axis, subsequently promoting HCC development (93). Another circRNA, hsa_circRNA_104348, showed opposite properties in relation to circLIFR discussed earlier. Indeed, hsa_circRNA_104348 can promote HCC progression, through the upregulation of the Wnt/β-catenin pathway, by sponging miR-187-3p (94).

## Conclusion

In this review, we discussed the involvement of circRNAs in the progression of NAFLD. Currently, this emerging class of circRNAs represents a key regulator of various biological and pathophysiological processes. Nonalcoholic fatty liver disease (NAFLD) is the most prevalent chronic liver disease and is strongly linked to the global epidemic of obesity and type 2 diabetes mellitus (T2DM), occurring in 70% of type-2 diabetes patients and in 80% of obese patients (95). Insulin resistance, leading to the development of prediabetes and its progression to T2DM, also results in a spontaneous onset of NAFLD (96). To date, there is no specific treatment for NAFLD. Current treatments paradigms, such as lifestyle improvements and weight loss, can help slow down disease progression but are not effective due to low patients’ compliance. Oftentime, anti-diabetics are used to treat NASH, but are not curative, uneffective for most patients and do not specifically target liver pathology (97). As the prevalence is rising, it becomes urgent to develop new therapies for NASH. In this review, we highlighted the important role of circRNAs affecting the different hallmarks of NAFLD, NASH and HCC: steatosis, autophagy, fibrosis, inflammation and oxidative stress (**Table 1**). The sponging effects of circRNAs on some miRs implicated in NASH onset and progression have shown their potential as therapeutic targets. Also, some existing treatments, such as metformin (anti-diabetic), have already shown their efficacy in the treatment of T2DM through the modification of non-coding RNAs expression (98). Thus, as we have discussed, circRNAs have an important role, although not yet fully understood, in the pathophysiology of NASH, highlighting the importance of developing specific treatments for NASH targeting circRNAs’ expression, in order to improve outcomes and halt disease progression. The emergence of technological advance, like high throughput sequencing, specific to circRNAs, will allow in the coming years a better understanding of their involvement in new biological processes.

**Table 1:** Implication of cirRNAs in liver diseases: NAFLD, NASH and hepatocellular carcinomas, and their effects.

Stage	Hallmark	Name	Target	Expression	Effect on disease progression	Ref
**NAFLD**	Lipid metabolism	Circ_Scd1	JAK2/STAT5	**+**	Exacerbates hepatic steatosis	(43)
		Circ_0000660	miR_693/IGFBP1	+	Reduces hepatic steatosis	(44)
	Autophagy	Circ_002581	miR_122/CPEB1/PTEN/AMPK/mTOR	–	Leads to autophagy dysfunction	(52)
**NASH**	Inflammation	Circ_RSF1	miR_146a-5p/Rac1/JNK/NFkB	+	Induces liver inflammation	(74)
		Circ_PSD3	Pro-inflammatories cytokines	–	Reduces cytokines production and liver inflammation	(59)
	Fibrosis		miR_92b-3p/SMAD7	+	Reduces liver fibrosis	(59)
		Circ_CREBBP	miR_1291/LEFTY2	+	Reduces liver fibrosis	(60)
		Circ_FBXW4	miR_18b-3p/TIMP1/PDGF/αSMA	–	Reduces liver fibrosis	(65)
		Circ_Ube2k	miR_149-5p/TGFβ2	+	Induces liver fibrosis	(66)
	Oxidative stress	Circ_Acc1	AMPK	+	Reduces hepatic oxidative stress	(79)
		Circ_021412	miR_1972/LPIN1	–	Reduces hepatic oxidative stress	(80, 81)
		Circ_0046366	miR_34a/PPARα	–	Reduces hepatic oxidative stress	(83, 84)
		Circ_0046367				
		Circ_SCAR	PGC1a/mROS	–	Reduces hepatic oxidative stress	(85)
		Circ_LIFR	miR_624-5p/β-catenin	–	Acts as a tumor suppressor	(89)
**HCC**		Circ_0005986	miR_129-5p/Notch1	+	Acts as a tumor suppressor	(90)
		Circ_TMEM45A	miR_65/IGF2	+	Promotes HCC progression	(92)
		Circ_GFRA1	miR_498/NAP1L3	+	Promotes HCC progression	(93)
		Circ_104348	miR_157-3p/β-catenin	+	Promotes HCC progression	(94)

## Author contributions

MY, J-BP and AD wrote the review. MP, KB, AG and AD edit the text. AD designed and edited all the review. All authors contributed to the article and approved the submitted version.

## Acknowledgments

Thank you to Fanny Krumm for her help for the visual abstract.

## Conflict of interest

J-BP, AG, and KB were employed by ILONOV. J-BP’s thesis is co-funded by an individual financial aid for training through research received by Ilonov from the State, through the Ministry in charge of Research.

The remaining authors declare that the research was conducted in the absence of any commercial or financial relationships that could be construed as a potential conflict of interest.

## Publisher’s note

All claims expressed in this article are solely those of the authors and do not necessarily represent those of their affiliated organizations, or those of the publisher, the editors and the reviewers. Any product that may be evaluated in this article, or claim that may be made by its manufacturer, is not guaranteed or endorsed by the publisher.

## Abbreviations

CCl4, Carbon tetrachloride; circRNA, circular RNA; HSC, Hepatic stellate cells; lncRNA, long non coding RNA; miR, micro RNA; mRNA, messenger RNA; mROS, Mitochondrial ROS; rRNA, ribosomal RNA; tRNA, transfer RNA.
